# Long non-coding RNA PITPNA-AS1 silencing suppresses proliferation, metastasis and epithelial-mesenchymal transition in non-small cell lung cancer cells by targeting microRNA-32-5p

**DOI:** 10.3892/mmr.2021.11911

**Published:** 2021-02-09

**Authors:** Gang Chen, Zhifeng Zheng, Junsheng Li, Peigang Zhang, Zhenjun Wang, Shiping Guo, Jun Ma, Jian Shen, Huixin Li

Mol Med Rep 23: 212, 2021; DOI: 10.3892/mmr.2021.11851

Following the publication of this article and after having solicited the opinions of all the participating authors, the Editorial Office was contacted by the authors to explain that the following changes are required to the list of contributing authors on the paper. The corresponding author of the article should be changed to the first author (Gang Chen), and a request was made that the name of the final author in the author list (Xiaotang Yang), who was also the original corresponding author, be removed. Therefore, the author affiliations and addresses, and the corresponding author information, in this paper have been revised as follows:

GANG CHEN^1^, ZHIFENG ZHENG^2^, JUNSHENG LI^3^, PEIGANG ZHANG^4^, ZHENJUN WANG^5^, SHIPING GUO^1^, JUN MA^6^, JIAN SHEN^7^ and HUIXIN LI^8^

^1^The Secondary Department of Thoracic Surgery, The Tumor Hospital Affiliated to Shanxi Medical University, Taiyuan, Shanxi 030013; ^2^Department of General Thoracic Surgery, Linfen People's Hospital, Linfen, Shanxi 041000; ^3^Department of Cardiothoracic Surgery, Taizhou Central Hospital (Taizhou University Hospital), Taizhou, Zhejiang 318000; ^4^Department of Cardio-Thoracic Surgery, The People's Hospital of Lvliang, Lvliang, Shanxi 033000; ^5^Cardiothoracic Surgery Department of Shanxi Yangquan Coal Industry (Group) Co., Ltd., Yangquan, Shanxi 045000; ^6^Department of Thoracic Surgery, Heji Hospital, Changzhi Medical College, Changzhi, Shanxi 046011; ^7^Department of Cardiothoracic Surgery, Changzhi People's Hospital Affiliated to Shanxi Medical University, Changzhi, Shanxi 046000; ^8^Department of Cardiothoracic Surgery, Yuci District People's Hospital, Jinzhong, Shanxi 030600, P.R. China

*Correspondence to*: Dr Gang Chen, The Secondary Department of Thoracic Surgery, The Tumor Hospital Affiliated to Shanxi Medical University, No. 3 of Workers' New Village, Xinghualing, Taiyuan, Shanxi 030013, P.R. China

E-mail: 1973chengang@163.com

Note that all the authors listed on the paper, including the author who has been removed, Xiaotang Yang, have agreed to this Corrigendum. The authors regret this error in the presentation of these affiliations, and apologize for any inconvenience caused.

